# Apolipoprotein E4, inhibitory network dysfunction, and Alzheimer’s disease

**DOI:** 10.1186/s13024-019-0324-6

**Published:** 2019-06-11

**Authors:** Ramsey Najm, Emily A. Jones, Yadong Huang

**Affiliations:** 1 Gladstone Institute of Neurological Disease, San Francisco, CA 94158 USA; 2 Developmental and Stem Cell Biology Graduate Program, University of California, San Francisco, CA 94143 USA; 3 Biomedical Sciences Graduate Program, University of California, San Francisco, CA 94143 USA; 4 Department of Neurology, University of California, San Francisco, CA 94143 USA; 5 Department of Pathology, University of California, San Francisco, CA 94143 USA

**Keywords:** Apolipoprotein E, Alzheimer’s disease, GABAergic interneuron, Hyperexcitability, Inhibitory network, Selective vulnerability, Tau

## Abstract

Apolipoprotein (apo) E4 is the major genetic risk factor for Alzheimer’s disease (AD), increasing risk and decreasing age of disease onset. Many studies have demonstrated the detrimental effects of apoE4 in varying cellular contexts. However, the underlying mechanisms explaining how apoE4 leads to cognitive decline are not fully understood. Recently, the combination of human induced pluripotent stem cell (hiPSC) modeling of neurological diseases in vitro and electrophysiological studies in vivo have begun to unravel the intersection between apoE4, neuronal subtype dysfunction or loss, subsequent network deficits, and eventual cognitive decline. In this review, we provide an overview of the literature describing apoE4’s detrimental effects in the central nervous system (CNS), specifically focusing on its contribution to neuronal subtype dysfunction or loss. We focus on γ-aminobutyric acid (GABA)-expressing interneurons in the hippocampus, which are selectively vulnerable to apoE4-mediated neurotoxicity. Additionally, we discuss the importance of the GABAergic inhibitory network to proper cognitive function and how dysfunction of this network manifests in AD. Finally, we examine how apoE4-mediated GABAergic interneuron loss can lead to inhibitory network deficits and how this deficit results in cognitive decline. We propose the following working model: Aging and/or stress induces neuronal expression of apoE. GABAergic interneurons are selectively vulnerable to intracellularly produced apoE4, through a tau dependent mechanism, which leads to their dysfunction and eventual death. In turn, GABAergic interneuron loss causes hyperexcitability and dysregulation of neural networks in the hippocampus and cortex. This dysfunction results in learning, memory, and other cognitive deficits that are the central features of AD.

## Background

Alzheimer’s disease (AD) is the most common form of dementia and is characterized by a progressive loss of memory and other cognitive functions [1–4]. Currently, there are 46.8 million people worldwide living with dementia, and this number is estimated to double every 20 years, reaching 74.7 million by 2030. Worldwide, AD cost $818 billion in 2015. By 2030, these costs could rise as high as $2 trillion [1]. This extreme expense combined with the growing aging population highlights the need for a better understanding of the disease mechanism and development of therapeutics.

AD is a multifactorial neurodegenerative disorder caused by interactions among multiple genetic and environmental factors. Mutations in three genes—those encoding amyloid precursor protein (APP), presenilin-1 (PS1), and presenilin-2 (PS2)—are linked to early-onset autosomal dominant AD, which accounts for less than 1% of all AD cases [2–4]. Apolipoprotein (apo) E4, an isoform of the *APOE* gene in humans, is the major genetic risk factor for late-onset familial and sporadic AD [4–8], which account for most AD cases. ApoE4 increases the risk and decreases the age of onset of AD in a gene dose dependent manner [4–11]. ApoE4 is present in roughly 20–25% of the human population, and apoE4 carriers account for 60–75% of AD cases in most clinical studies [11], highlighting the importance of apoE4 in AD pathogenesis.

AD is characterized by two molecular pathological hallmarks: extracellular amyloid-β (Aβ) plaques and intracellular neurofibrillary tangles (NFTs) [2–4]. The accumulation of Aβ plaques and NFTs is associated with significant neuronal and synaptic loss as well as neuroinflammation. Both of these pathologies are exacerbated by the presence of apoE4 [4–7, 12]. Biochemical, cellular, transgenic animal, and clinical studies have suggested many potential explanations for apoE4’s contribution to AD pathogenesis [4–7, 12]. This review focuses on apoE4’s detrimental effects on GABAergic interneurons, the network deficits resulting from GABAergic interneuron dysfunction or loss, and the mechanisms that link these deficits to AD pathogenesis and cognitive decline.

### ApoE structure, function, and expression in the CNS

**Fig. 1:**
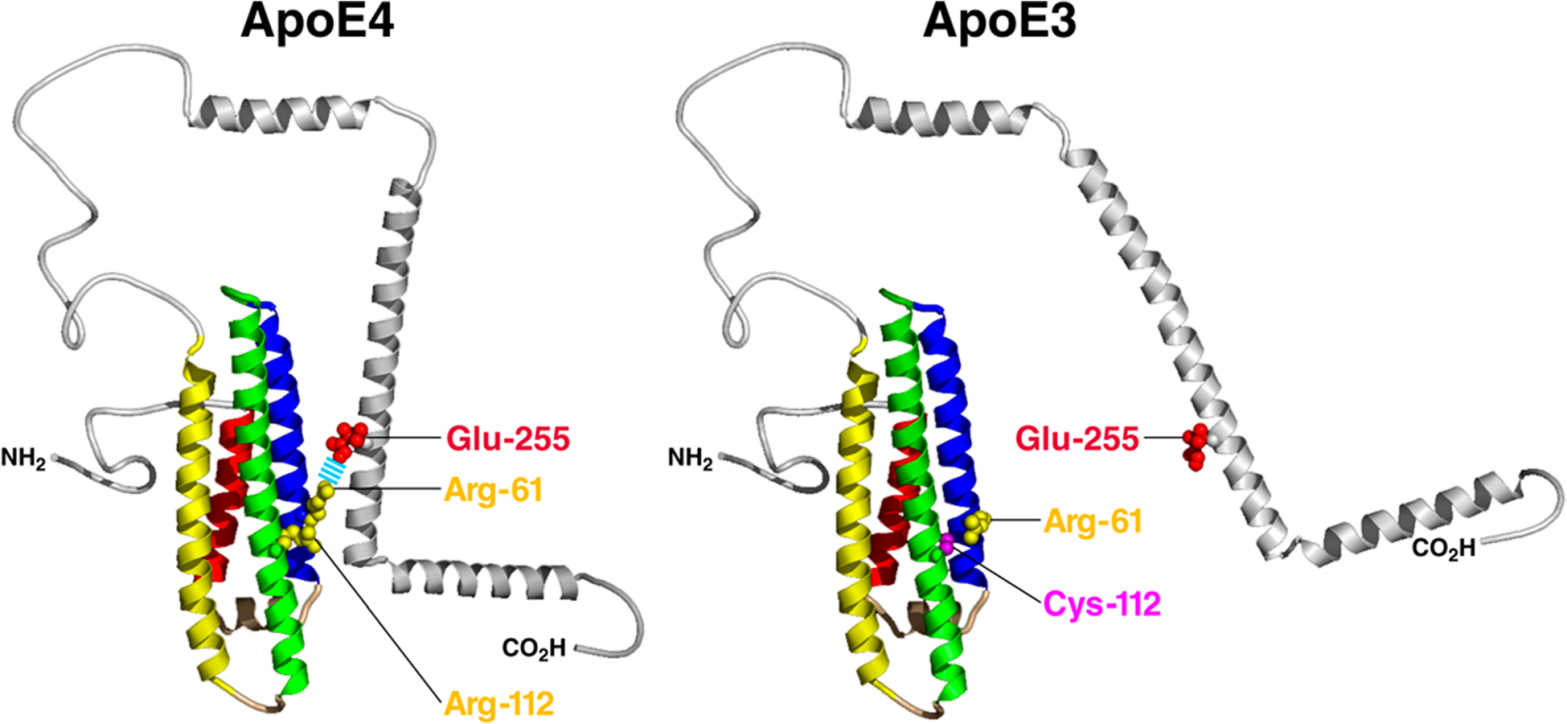
Model of domain interaction as a determinant of conformation of apoE. In apoE4 *(left)*, Arg-112 orients the side chain of Arg-61 into the aqueous environment where it can interact with Glu-255, resulting in interaction between the amino- and carboxyl-terminal domains. In apoE3 *(right)*, Arg-61 is not available to interact with residues in the carboxyl-terminal domain, resulting in a very different overall conformation

ApoE is a 34-kDa protein comprised of 299 amino acids. It is a polymorphic protein with three common isoforms, apoE2, apoE3, and apoE4 in humans. Each isoform differs only by one or two amino acids [4, 6, 8, 13, 14]. The apoE3 and apoE4 amino acid sequences differ only at position 112 where apoE4 has an Arg instead of a Cys. This seemingly small difference induces significant changes to its structures and biological functions. ApoE is comprised of two domains: the amino-terminal domain and carboxyl-terminal domain. These two domains contain the receptor-binding region and the lipid-binding region, respectively, and are joined by a flexible hinge region. Multiple research groups have investigated potential interaction between the two domains, which is important to apoE’s function [15–17]. Nuclear magnetic resonance (NMR) analysis of a monomeric mutant form of apoE3 recently revealed a potential full-length structure of apoE. In this monomeric mutant apoE3, Arg-61 interacts with Thr-194 via a H-bond and Lys-95 forms a salt bridge with Glu-255 [17]. Whether this mutant form of apoE3 truthfully reflects the biophysical and biological properties of wildtype apoE3 needs to be further evaluated. An alternative model which used X-ray crystallography and circular dichroism spectroscopy to identify the structure of the amino-terminus and the carboxyl-terminus, respectively, demonstrates that Arg-112 in apoE4 interacts with Glu-109, exposing Arg-61 to interact with Glu-255. This domain interaction mediated by a salt bridge formation between Arg-61 and Glu-255 is unique to apoE4 (Fig. 1) [15]. This model of apoE4 domain interaction has been supported by Fluorescence Resonance Energy Transfer and electron paramagnetic resonance tests [18] and was observed in live neurons expressing apoE4 [19]. Importantly, this domain interaction renders apoE4 to be more susceptible to proteolytic cleavage, resulting in the generation of neurotoxic apoE4 fragments [20–22].

Initially, apoE was described as a lipid transport protein and was shown to play a key role in cholesterol metabolism and cardiovascular disease. However, by the mid-1980s, it had become apparent that apoE also plays significant roles in neuronal repair and remodeling as well as in neurological disease [8, 12, 13]. Astrocytes are the primary source of apoE in the brain [23, 24]. However, under aging and stress conditions, neurons also produce apoE, albeit at lower levels than astrocytes [25, 26]. Microglia also express apoE, especially under conditions of neurodegeneration and/or inflammation, and the interplay between apoE and microglia has been reviewed elsewhere [27]. Cellular origin plays a crucial role in apoE’s biophysical properties and pathological effects. Astrocytic apoE might be more heavily involved in Aβ pathology, while neuronal apoE has been shown to be more impactful on neuronal function and survival as well as on NFT formation. Clearly, more research needs to be done to completely understand how cellular origin affects apoE’s biological and pathological characteristics [27, 28]. Overall, it has been demonstrated, both in vivo and in vitro, that apoE plays major roles in AD pathogenesis in both an Aβ-dependent and independent manner, and different cellular sources of apoE4 may contribute in distinct ways to AD pathogenesis [4–8, 12–14, 21, 22].

### Aβ-dependent roles of ApoE4 in AD

Accumulation of fibrillar Aβ peptides (amyloid plaques) in the brain is a requirement for an AD pathological diagnosis. Aβ accumulation can take place due to an imbalance between production and clearance in the brain. ApoE is associated with amyloid plaques and its roles in Aβ-related pathologies have been extensively reviewed elsewhere [5, 7, 29–32]. Here we only briefly overview its relationship to Aβ aggregation/deposition and clearance in the brain.

#### ApoE4 and Aβ seeding, aggregation, and plaque formation

The roles of apoE in Aβ seeding, aggregation, and plaque formation are still not fully understood, as research groups have shown that both increasing or decreasing apoE levels reduces plaque load [5, 7, 33–48]. These seemingly conflicting results are most likely due to the model in question, the complexity of apoE biology, and the cellular source of apoE, as lipidation status, isoform, cell source, expression level, and the aggressiveness of the Aβ production in the model can complicate results. For example, increasing apoE levels in the brain has been shown to suppress Aβ deposition, facilitating Aβ clearance, and reverse memory deficits [49–51]. However, these results were disputed by several follow-up studies. Notably, genetically decreasing apoE expression results in less Aβ deposition in amyloid mouse models, independently of apoE isoform [39, 40]. Reducing apoE through immunotherapy has also been shown to significantly reduce insoluble Aβ levels [52]. ApoE4 has also been shown to facilitate Aβ production in vitro [53]; thus, lowering apoE4 may decrease Aβ production.

Furthermore, recent studies have demonstrated that increasing or decreasing apoE levels at specific time points during Aβ plaque formation differentially affects Aβ plaque associated pathology. In an APP/PS1 mouse model where human apoE3 or apoE4 is expressed exclusively in astrocytes, apoE4 accelerated amyloid pathology. More specifically, increased expression of astrocytic apoE4 during the early seeding stage of amyloid plaque formation increased amyloid deposition and neuronal pathology [54]. In APP/PS1–21 mice with either the human apoE3 or apoE4 allele homozygously knocked-in (apoE-KI), apoE levels were reduced at different ages using antisense oligonucleotides (ASO) in order to better understand how the timing of apoE expression impacts Aβ accumulation and pathology. ASO treatment directly after birth led to a significant decrease in Aβ pathology opposed to treatment starting at 6-weeks of age (when significant amyloidosis has occurred due to the aggressive nature of amyloid pathology in these mice). Lowering apoE4 levels at 6-weeks of age led to an increase in Aβ plaque size and reduction in plaque-associated neuritic dystrophy with no change in overall plaque load [55]. Taken together, these results indicate that apoE plays a significant role in the initiation of Aβ pathology; however, after Aβ pathology has been initiated, lowering apoE modulates plaque size and toxicity.

#### ApoE4 and Aβ clearance

The role that apoE plays in clearing Aβ has been heavily investigated as well [29, 30, 32, 56–60]. Multiple pathways exist to clear Aβ, including proteolytic degradation, cellular clearance, and the cerebrovascular clearance, all of which have been reviewed elsewhere [7]. It has been suggested that apoE facilitates Aβ degradation by converting its structure into one that is more recognizable by proteolytic enzymes. ApoE assists in Aβ clearance in an isoform-dependent manner wherein apoE2 > apoE3 > apoE4 [29, 30, 59]. Strikingly, C-terminally truncated apoE4 clears Aβ inefficiently and acts in concert with Aβ to elicit neuronal and behavioral deficits in transgenic mice [61]. Astrocytes have been shown to internalize and degrade Aβ in an apoE dependent manner [31]. ApoE also promotes Aβ clearance by activating phagocytosis and migration of microglia wherein apoE3 is more effective than apoE4 [62–64]. Astrocytic apoE4 significantly increases brain Aβ half-life relative to apoE3*,* suggesting an impairment of Aβ clearance by astrocytic apoE4 relative to apoE3 [54, 65]. In addition to astrocytes, neurons are also capable of up taking and degrading Aβ, however more work needs to be done in order to dissect the mechanism of Aβ clearance in neurons [66]. Although apoE interacts with amyloid, it should be noted that plaque load does not correlate well with cognitive impairments, highlighted most obviously by people with substantial plaque burdens and normal cognition [67]. Therefore, it is important to also consider apoE4’s roles in AD pathology independently of Aβ as well.

### Aβ-independent roles of ApoE4 in AD

ApoE4 has also been shown to affect many different pathological processes independently of Aβ. For example, both apoE4 transgenic and apoE4-KI mice show age- and sex-dependent learning and memory deficits in the absence of Aβ accumulation, as compared to apoE3 transgenic and apoE3-KI mice [68–71]. ApoE4 impairs synaptogenesis and decreases dendritic spine density in vivo and in vitro in primary neuronal cultures [72, 73]. Furthermore, it impairs adult hippocampal neurogenesis in mice and affects cortical thickness, brain activity, and mitochondrial function well before significant Aβ accumulation in the human brains [74–76]. Other non-amyloid pathways affected by apoE4 include lipid metabolism, synaptic plasticity, and most relevant to AD, tau pathology [77].

In response to injury, or stress such as normal aging, neurons express apoE, likely to facilitate transport of cholesterol and other lipids for membrane repair and/or remodeling [22]. As mentioned above, apoE4 is highly susceptible to neuron-specific proteolysis, which generates neurotoxic fragments [20, 77–79]. These fragments escape the secretory pathway and enter the cytosol, where they stimulate tau-phosphorylation and interact with mitochondria, leading to mitochondrial dysfunction and neurodegeneration [4]. In particular, GABAergic interneurons in the dentate gyrus (DG) are particularly vulnerable to apoE4 fragment-mediated neurotoxicity, and in apoE4 fragment transgenic mice, knocking out tau rescues GABAergic interneuron loss as well as learning and memory deficits, demonstrating the tau-dependent nature of apoE4-induced cognitive impairment [71]. Therefore, in order to better understand apoE4’s pathophysiology in the context of AD, it is important to study its interaction and impact on tau.

In mutant human Tau-P301S transgenic mice, expression of apoE4 led to more advanced tau pathology, brain atrophy, and neuroinflammation. Interestingly, knocking-out apoE (apoE-KO) protected the mice from Tau-P301S-induced neurodegeneration and neuroinflammation. These data strongly support apoE4’s gain of toxic effects on tau pathology and its related neurodegeneration and neuroinflammation, all of which are independent of Aβ [80]. However, a recent study using a gene delivery approach, in which adeno-associated virus (AAV) expressing human tau protein containing the P301L mutation (AAV-Tau^P301L^) was injected into the cerebral lateral ventricles of neonatal apoE2-KI, apoE3-KI, and apoE4-KI mice, resulted in contradictory findings. Specifically, 6-month old apoE2-KI mice injected with the AAV-Tau^P301L^ construct at postnatal day 0 had significantly higher levels of hyperphosphorylated and aggregated tau, as well as more severe behavioral abnormalities than did 6-month old apoE3-KI and apoE4-KI mice under the same conditions [81]. Strikingly, in humans, *APOE2* is associated with increased risk of two tauopathies: progressive supranuclear palsy and corticobasal degeneration [81]. The discrepancies between these studies could be the result of differences between model systems, such as cell type specificity and the overall level of tau expression, differences in toxicity between Tau-P301S and Tau-P301L mutations, and age of the mice. It should be noted that clinical manifestations of Tau-P301S and Tau-P301L are related to frontotemporal dementia (FTD) but not AD. In order to fully understand apoE isoform-dependent role in tau pathology in the context of AD, more in-depth research and new animal models are required.

### Inhibitory system dysfunction in AD

In recent years, it has become clear that neurodegenerative diseases target specific neuronal populations [82]. GABAergic interneuron dysfunction, in particular, is found in a range of neurological and psychiatric disorders, including schizophrenia, autism, Fragile X syndrome, epilepsy, migraines, depression, bipolar disorder, and AD [83]. Loss of GABA, the primary inhibitory neurotransmitter in the brain, is a key component of AD. Post-mortem tissue from AD patients shows reduced GABA level throughout the brain, particularly in temporal, parietal, and frontal cortices [84, 85]. Post-mortem cortices from AD patients contain reduced GABAergic terminals, particularly near amyloid plaques [86, 87]. AD patients show reduced cortical GABA as measured by positron emission tomography, especially in the temporal cortex [88, 89], and reduced GABA in cerebrospinal fluid [90–93]. Additionally, AD patients have a specific loss of somatostatin-positive interneurons in the cortex [94] and hippocampus [95]. Several other neuronal subtypes are also affected by AD pathology, including cholinergic and glutamatergic neurons, whose loss and dysfunction in turn contribute to cognitive impairment [96]. This review will focus on the consequences of GABAergic interneuron loss and dysfunction, which have broad consequences at the network and behavioral level.

Loss of GABA and GABAergic interneurons in AD patients may be responsible for network hyperactivity manifesting as seizures. Substantial evidence shows that loss of GABAergic tone leads to seizures [97]. 10–22% of AD patients exhibit seizures [98–100], as do hAPP_FAD_ mice [101], and the onset of these seizures precedes cognitive decline [102]. Levetiracetam, an anti-epileptic drug, successfully reverses hyperexcitability and learning and memory deficits in an hAPP_FAD_ mouse model of AD [103, 104] and in aged mice [105–107]. Cognitively normal elderly, amnestic mild cognitive impairment (MCI), and AD patients all show cognitive improvement following chronic levetiracetam administration [108–110]. Thus, GABAergic dysfunction contributes to network-wide deficits in AD, which may in turn harm cognition.

GABAergic inhibitory interneurons make up a minority of neurons within the brain but play an outsized role in coordinating activity [111]. Inhibitory interneurons regulate network oscillations, which synchronize neuronal activity to rhythms that are crucial to learning and memory [112–116]. Inhibition also prevents hyperactivity of excitatory principal cells, which disrupts normally sparse neural coding and leads to decreased signal-to-noise ratio [117–119]. Furthermore, reducing hippocampal GABA levels impairs learning and memory [120, 121], and silencing inhibitory interneurons in the dentate gyrus prevents both encoding of new memories and recall of old memories [122]. Given their importance to proper learning and memory, it is crucial to better understand GABAergic inhibitory interneuron dysfunction and/or loss in the context of AD. As apoE4 is the major genetic risk factor for AD, understanding its effect on GABAergic interneurons, a population that is particularly vulnerable to apoE4 pathology, is essential. ApoE is expressed in neurons during periods of stress or normal aging. The neuronally expressed apoE4 is more susceptible to proteolytic cleavage and cytotoxic fragment generation. In the following sections, evidence for GABAergic interneuron susceptibility to apoE4 and the subsequent network deficits that result of inhibitory neuron loss, culminating in learning and memory deficits will be discussed.

### GABAergic interneuron susceptibility to ApoE4

#### In vivo studies

Many lines of evidence from in vivo studies contribute to the hypothesis that GABAergic interneurons in the hippocampus are disproportionately susceptible to apoE4-mediated toxicity. For example, apoE4-KI mice display an age- and tau-dependent decrease in hilar GABAergic somatostatin-positive interneurons in the hippocampus [71]. The extent of this inhibitory interneuron loss correlates with both decreased adult hippocampal neurogenesis and with learning and memory deficits [70, 74]. The adverse effects of apoE4 are prevented by tau removal, indicating a direct link between tau pathology, apoE4, and GABAergic interneuron death [71]. Interestingly, the cellular source of apoE is critical to its pathological effect on GABAergic interneurons. ApoE4 undergoes proteolytic cleavage which generates neurotoxic fragments only when produced in neurons, but not when produced in astrocytes [20]. When expressed in neurons, apoE3 is excitoprotective whereas apoE4 is not; however, when expressed in astrocytes, apoE3 and apoE4 are equally excitoprotective [123]. Likewise, when expressed in neurons, apoE4 decreases dendrite arborization and spine density whereas apoE4 expressed in astrocytes does not show similar effects [124]. Importantly, deletion of apoE4 in GABAergic interneurons, but not deletion of apoE4 in astrocytes, is sufficient to protect aged mice from apoE4-induced GABAergic interneuron loss and learning and memory deficits [125]. These findings suggest that, although the majority of apoE is produced in astrocytes, it is apoE4 produced within GABAergic interneurons that is detrimental to their survival in vivo which leads to deficits in both learning and memory in AD models. Strikingly, bolstering inhibitory function, either through systemic GABA-agonist treatment [126] or through transplant of mouse derived inhibitory interneuron progenitors directly into the hippocampus [127], restores learning and memory in aged apoE4-KI mice without or with mutant hAPP_FAD_ expression.

#### In vitro studies

GABAergic interneuron selective vulnerability to apoE4 is also supported by a recent study in an in vitro model using hiPSC-derived neurons with different *APOE* genotypes [128]. These included *APOE4, APOE3,* gene-edited isogenic *APOE3* derived from *APOE4,* and *APOE*-deficient hiPSC lines. Strikingly, much of AD pathology seen in vivo was successfully recapitulated in this hiPSC-derived neuronal model in vitro*.* For example, apoE4/4 neurons produced significantly more Aβ and phosphorylated tau than apoE3/3 neurons. ApoE4/4 GABAergic interneurons in particular showed degeneration and displayed significantly elevated phosphorylated tau levels compared to apoE3/3 GABAergic interneurons. Importantly, there was no significant loss of glutamatergic neurons and dopaminergic neurons in apoE4/4 hiPSC-derived neuron cultures, suggesting a preferential detrimental effect of apoE4 on GABAergic neurons. Converting *APOE4* to *APOE3* by gene editing rescued these pathologies, including tau hyperphosphorylation, Aβ_40_ and Aβ_42_ overproduction, and GABAergic interneuron loss, suggesting that neuronal apoE4 expression alone was sufficient to induce these interneuron pathologies. Finally, a small molecule that renders apoE4 ‘apoE3-like’ by changing the protein’s conformation to nullify apoE4’s unique domain interaction was tested. Treatment with this structure corrector significantly decreased apoE4 fragmentation, reduced the levels of hyperphosphorylated tau and Aβ_40_ or Aβ_42_ overproduction and/or secretion, and increased GABAergic interneuron survival [128], again suggesting that the specific actions of neuronal apoE are responsible for this GABAergic interneuron specific toxicity. Isogenic hiPSC lines with an apoE3/3 or apoE4/4 genotype have also been used to study transcriptomic, molecular, and cellular alterations caused by apoE4 [129]. In hiPSC-derived isogenic *APOE4* neurons, genes known to control synaptic function were significantly downregulated, there was an increase in Aβ_42_ secretion, and an increase in hyperphosphorylated tau levels in isogenic *APOE4* neurons versus *APOE3* controls [129].

### ApoE4-mediated GABAergic interneuron loss and inhibitory network dysfunction in AD

Given that hippocampal GABAergic interneurons are selectively vulnerable to apoE4, an intriguing question is: how does interneuron dysfunction manifest at the network and behavioral or clinical levels? At the network level, loss of GABAergic function can lead to deficits in both tonic and phasic inhibition. Loss of tonic inhibition manifests itself most prominently in AD patients as hypersynchrony, leading to epilepsy and olfactory processing deficits, as well as hyperactivity, leading to aberrantly increased activation of cortical and hippocampal networks [130]. Loss of phasic inhibition manifests as reduced hippocampal rhythms [130]. These network consequences of inhibitory deficits each contribute to learning and memory impairments [131]. The following sections will address these manifestations of inhibitory network dysfunction that occur as a result of apoE4 expression.

#### ApoE4 and GABAergic interneuron dysfunction leading to seizure activity in AD

The loss of GABA and GABAergic interneurons in AD patients may lead to network hyperactivity, most commonly observed through seizures. ApoE4 carriers have a higher risk [132–136] and earlier onset [137–139] of developing idiopathic or secondary temporal lobe epilepsy. It is still unclear whether these patients demonstrate a higher risk for developing AD later in life, or if indeed the proportion of AD patients with concomitant epilepsy is enriched for apoE4 carriers. In addition to increased risk, apoE4 is also associated with increased epileptic pathology. The presence of apoE4 is correlated with smaller neuron size and increased DNA damage in temporal lobes of epilepsy patients [140], and epilepsy patients with at least one *APOE4* allele are six times more likely to exhibit treatment resistance [141]. Investigating the connection between apoE4 and epilepsy may shed light on its role in large-scale network dysfunction in AD.

#### ApoE4-mediated GABAergic interneuron dysfunction and olfactory deficits in AD

Olfactory dysfunction is also an early and common symptom of AD as well as a result of carrying apoE4 and odor identification ability predicts future cognitive decline [142–145], making olfactory acuity a potential early signal of underlying neurodegenerative processes. ApoE4 carriers show particularly marked deficits in odor identification and memory relative to non-carriers [146], and evidence suggests disrupted GABA signaling in the olfactory bulb may mediate this olfactory loss [147]. In vivo electrophysiological recordings from aged apoE4-KI mice with odor memory deficits revealed increased local field potential response to odors in both the olfactory bulb and in primary olfactory cortex [148], which was attributed to inhibitory dysfunction. These studies together suggest that apoE4-mediated odor memory impairment, a potential early biomarker of cognitive dysfunction, may be due to apoE4-induced hyperactivity.

#### ApoE4 and microglial dysfunction in the GABAergic inhibitory network and AD

The link between apoE, microglia, and GABAergic interneuron dysfunction is also an emerging area of interest in the context of network dysfunction and AD. ApoE expression in microglia and its roles in microglial physiology and pathology have recently been actively explored. ApoE is upregulated in primed/activated microglia [149, 150], and apoE signaling in microglia following phagocytosis of apoptotic neurons or in response to Aβ accumulation leads to a transcriptional switch from promoting homeostasis to promoting inflammation and neurodegeneration [150, 151]. Deletion of the *Apoe* gene suppresses microglial activation in response to Aβ accumulation and prevents migration of microglia toward amyloid plaques [150]. However, the effect of specific apoE isoforms has yet to be explored [152, 153]. It has been reported that activated microglia migrate to inhibitory synapses and displace them from excitatory neurons [154] and an increase in CX3CR1 expression in activated microglia suppresses GABA_A_ receptor signaling in excitatory neurons [155], both of which could contribute to GABAergic inhibitory network deficits in the context of apoE4. Another avenue by which microglial dysfunction may affect GABAergic interneurons is through perineuronal nets. Perineuronal nets are extracellular matrix structures which surround synapses of highly active neuronal subtypes and are associated with microglia [156]. These structures are involved in synapse development, stabilization and remodeling, buffering ions, and regulating the synapse microenvironment [157]. AD patients have reduced perineuronal net density [158]. Strikingly, the majority of neurons surrounded by perineuronal nets are parvalbumin-expressing GABAergic interneurons [159], and these interneurons show deficits in perineuronal net density in AD model of mice [160]. Since perineuronal nets protect these interneurons from oxidative stress and other injuries [161], it is possible that their breakdown in AD, which can be triggered or exacerbated by microglial dysfunction, may lead to interneuron dysfunction or death and thus inhibitory network deficits.

#### ApoE4 and network hyperactivity induced by GABAergic interneuron dysfunction

Network hyperactivity is an overarching symptom of AD and is evident in human apoE4 carriers. More specifically, hyperactivity in two networks which are normally disengaged during task performance in healthy individuals has been demonstrated by multiple groups. First, cognitively normal apoE4 carriers show reduced task-induced deactivation of the default mode network (DMN) [162–164]. Higher resting state GABA levels in the DMN are associated with enhanced task-induced deactivation of this network [165–167], suggesting that this DMN hyperactivity could be the result of inhibitory deficits. Reduced ability to deactivate the DMN during memory encoding is found in AD patients [168–170] and is correlated with worse task performance [171], linking this apoE4-induced deficit to memory impairments. Second, healthy elderly apoE4 carriers show increased hippocampal and entorhinal activation during encoding task performance [172, 173]. A recent study found that aged apoE4-KI mice had increased field potential synchrony and pyramidal cell firing in the entorhinal cortex [174]. This activation is dysfunctional hyperactivity rather than task-related, as levetiracetam treatment of amnestic MCI patients both reduces hippocampal over-activation and improves cognitive performance during a recognition memory task [109, 175]. Greater hippocampal activation during encoding tasks is associated with worse task performance [176] in MCI and AD patients [172, 177], and even predicts future cognitive decline in cognitively healthy elderly [178]. Finally, aberrant activity increases in these networks are seen even prior to aging. Healthy young and middle-aged adult apoE4 carriers show increased DMN activation at rest [179] and increased hippocampal activation during encoding task performance [179–181], suggesting that apoE4-induced network hyperactivity occurs before significant Aβ accumulation in human brains.

#### ApoE4-mediated GABAergic interneuron loss and hippocampal network dysfunction and memory deficits

Susceptibility of GABAergic interneurons to apoE4 and subsequent loss of inhibitory function can also lead to reduced coordination of hippocampal network activity involved in memory. ApoE4-KI mice show reduced abundance of sharp-wave ripples, the local field potential of hippocampal replay events which are critical for consolidating spatial memory [182, 183]. ApoE4-KI mice also display reduced slow gamma power throughout the hippocampal circuit during ripple events, suggesting reduced accuracy of these replay events [182, 184]. Thus, apoE4 leads to reduced instances as well as accuracy of spatial memory consolidation. Notably, removing apoE4 from inhibitory interneurons specifically rescues slow gamma power and learning and memory deficits, indicating that these phenotypes are caused by intraneuronal apoE4 expressed in GABAergic interneurons. Younger mice recorded before the onset of significant interneuron loss do not show significant slow gamma power loss, further implicating inhibitory interneurons in apoE4-induced hippocampal gamma loss [182].

### Conclusions and perspectives

#### Conclusion: working model of ApoE4-induced GABAergic interneuron deficit and network dysfunction in AD

**Fig. 2:**
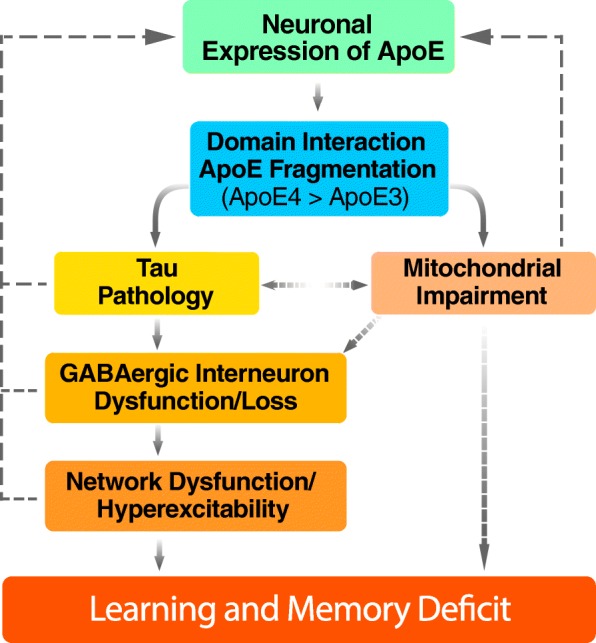
Proposed working model of apoE4-induced GABAergic interneuron deficit and network dysfunction in AD. In response to aging, stress, or injury, apoE is expressed in neurons to facilitate neuronal repair and remodeling. However, higher apoE4 fragmentation due to its pathological conformation (domain interaction) leads to tau pathology and mitochondrial impairments. GABAergic interneurons in the hippocampus are selectively vulnerable to apoE4 toxicity, resulting in dysfunction and eventual loss. The inhibitory interneuron loss leads to network dysfunction and hyperexcitability, resulting in a positive feedback loop culminating in learning and memory deficits

The combination of the data presented above paints a more complete picture of the mechanism underlying apoE4 mediated cognitive decline. We present a model wherein injury or aging-related stress induces neuronal apoE expression. Due to its pathological conformation (domain interaction), apoE4 is more susceptible to proteolytic cleavage than apoE3, leading to increased levels of neurotoxic fragment generation, and through a tau-dependent mechanism, results in GABAergic interneuron dysfunction and death. The loss of hippocampal GABAergic interneurons leads to network dysfunction and hyperexcitability. The network dysfunction and hyperexcitability themselves contribute to learning and memory deficits as well as induce further stress, and therefore more neuronal expression of apoE. This process culminates in further GABAergic interneuron loss and eventual cognitive decline (Fig. 2).

It is apparent that more research needs to be done on understanding apoE4’s roles in AD pathogenesis and on developing therapeutics targeted to its specific detrimental effects. This can be achieved by focusing on: 1) better understanding of the selective vulnerability of GABAergic interneurons to apoE4 and 2) better therapeutic approaches addressing apoE4’s detrimental effects at a molecular, cellular, and network level.

#### Perspective: better understanding of the selective vulnerability of GABAergic interneurons to ApoE4

Based on both in vivo and in vitro studies, GABAergic interneurons appear to be selectively vulnerable to apoE4 induced neurotoxicity, although the underlying molecular and cellular mechanisms are still unclear. However, a number of potential hypotheses can be put forth for experimental testing [82]. While many potential pathways could cause GABAergic interneurons to be selectively vulnerable to apoE4, we would suggest focusing on the following two. One hypothesis is that GABAergic interneurons might generate more neurotoxic apoE4 fragments due to higher expression of apoE or its cleaving protease. This increased fragment generation would lead to increased neurotoxicity and cell death [20, 123–125]. Upon identification of the apoE4 cleaving protease, a testable hypothesis would be to investigate whether GABAergic interneurons produce more of this protease and therefore generate more neurotoxic apoE4 fragments leading to their death. A second hypothesis is that the metabolic demand of GABAergic interneurons makes them selectively vulnerable to apoE4 pathology. Multiple groups have presented evidence of mitochondrial impairments in AD [185, 186]. As mentioned previously, apoE4 induces deficits in mitochondrial function [187, 188]. Interestingly, there is increasing evidence that GABAergic interneurons require a unique level of high-energy expenditure [189]. An intriguing explanation for GABAergic interneuron selective vulnerability to apoE4, then, is that they have unique demands for high energy production which, in turn, makes them vulnerable to any perturbation of mitochondrial function [189, 190]. A recent study reports that apoE4-expressing neuronal cells have 50% less reserve capacity to generate ATP than apoE3-expressing neuronal cells as well as widespread changes in mitochondrial protein production and translocation, which makes apoE4-expressing neuronal cells more vulnerable to metabolic stress [191]. Building off these data, a testable hypothesis is that apoE4-induced mitochondrial dysfunction is especially damaging to GABAergic interneurons because of their especially high demands for metabolic energy.

#### Perspective: better therapies targeting ApoE4’s detrimental effects on GABAergic interneurons

Several approaches could be further developed for treating apoE4-mediated pathologies or GABAergic dysfunction. First, apoE4-mediated GABAergic deficits and cognitive decline could be treated with small molecules. For example, treating apoE4-KI mice with pentobarbital early in life prevents learning and memory deficits late in life [126]. Furthermore, the use of a structure corrector has been shown in vitro to ameliorate apoE4-mediated AD pathologies in hiPSC-derived neurons, including GABAergic neuron deficits [128]. However, developing new drugs for new targets can be prohibitively expensive. Using current screening methods it is possible to find combinations of existing drugs (drug repurposing) that can correct pathological phenotypes of AD [192, 193]. In the context of apoE4, it would be especially interesting to identify existing drugs that can enhance GABAergic interneuron function or can correct gene expression signatures in apoE4/4 neurons to a more ‘apoE3/3-like’ profile.

Several treatments which enhance inhibition have been tested in animal models and in clinical trials. GABA_A_ receptor potentiators or agonists ameliorate apoE4- or amyloid-induced toxicity and improve cognition in rodent models of AD and normal aging [126, 194]. However, across several clinical trials, these agents have produced behavioral, but not cognitive, improvements [85]. Unfortunately, these therapeutics produce undesirable side effects which limit long-term use [195, 196]. Anti-epileptic agents similarly show promise in animal models [103, 104], but have not produced cognitive improvements in clinical trials [85], with the exception of levetiracetam that improved cognition and reduced hippocampal hyperactivity in preclinical and initial clinical studies [107–110, 175, 197–199]. However, trials for both of these therapeutics used only small cohorts over short treatment periods, so further study in larger clinical trials is required. Moreover, specifically targeted therapies might be more beneficial. For instance, theta burst stimulation via transcranial magnetic stimulation has been used successfully to increase GABA within the DMN [200]. This could be used to rescue specific network pathologies rather than globally increasing inhibition.

Driving specific interneuron populations could be used to rescue network synchrony. Two foundational optogenetic studies demonstrated that optogenetically driving inhibitory interneurons specifically enhances slow gamma frequency oscillations throughout cortex, reducing circuit noise while amplifying circuit signal [201, 202]. Non-invasive stimulation can augment endogenous network oscillations to enhance learning and memory. In humans, transcranial magnetic stimulation enhances cortical slow waves and thus improve task performance [203]. In mice, slow gamma frequency visual or audio input entrains neural firing to this frequency in the cortex and hippocampus and reduces Aβ pathology and microglial abnormalities [204, 205]. Finally, enhancing activity of existing interneurons could also attenuate the network effects. For example, exogenous neuregulin 1 increases excitability of parvalbumin-positive interneurons [206] and has been used to restore hippocampal theta synchrony and fear conditioning in a mouse model of schizophrenia, which showed inhibitory impairments [207].

In addition to targeting susceptibility of GABAergic interneurons to apoE4 and the subsequent network hyperexcitability that results from inhibitory neuron loss, another potential therapy is to replace the lost population of GABAergic interneurons. Cell replacement therapy has been explored in the context of various neurodegenerative diseases [208–211]. Notably, it has been shown that GABAergic interneuron progenitor transplantation has potential to be an effective method to correct seizure activity in an epilepsy model [212]. Likewise, transplantation of mouse MGE-derived GABAergic progenitors into aged apoE4-KI mice without or with Aβ accumulation rescues learning and memory deficits [127]. Furthermore, transplanting Nav1.1-overexpressing interneurons derived from the mouse MGE into an hAPP_FAD_ mouse model enhances behavior-dependent gamma oscillatory activity, reduces network hypersynchrony, and improves cognitive function [213]. In the future, it would be interesting to employ a similar cell therapeutic strategy, using hiPSC-derived GABAergic progenitors with an apoE3/3 genotype as donor cells for transplantation, to treat hyperexcitability and network deficits in an apoE4 model of AD.

Clearly, new hope for effective therapeutics of AD relies upon the ability of scientists to explore multiple lines of inquiry. Moving forward, it is certainly conceivable that there will be combination therapies implemented, with drugs targeting Aβ, tau, inflammation, apoE4, and apoE4-induced GABAergic interneuron impairment.

## Data Availability

Not applicable.

## Abbreviations

AAV: Adeno-associated virus
AD: Alzheimer’s disease
Apo: Apolipoprotein
apoE-KI: ApoE knock-in
apoE-KO: ApoE knock-out
APP: Amyloid precursor protein
ASO: Antisense oligonucleotides
Aβ: Amyloid-β
CNS: Central nervous system
DMN: Default mode network
GABA: γ-aminobutyric acid
hiPSC: Human induced pluripotent stem cell
hPSC: Human pluripotent stem cell
MCI: Mild cognitive impairment
MGE: Medial ganglionic eminence
NFTs: Neurofibrillary tangles
NMR: Nuclear magnetic resonance
PS1: Presenilin-1
PS2: Presenilin-2
